# Sustainability and normalization of an intervention to improve evidence-based myocardial infarction care in Tanzania

**DOI:** 10.21203/rs.3.rs-7368551/v1

**Published:** 2025-08-29

**Authors:** Claire Wang, Francis M Sakita, Spencer Sumner, Frida M Shayo, Zebadia Martin, Winnie Msangi, James J Munisi, Elly Mulesi, Ayshat M Aboud, Janet P Bettger, Hayden B Bosworth, Julian T Hertz

**Affiliations:** Duke University School of Medicine 0009-0000-6543-9768; Kilimanjaro Christian Medical Centre; Duke University School of Medicine; Kilimanjaro Christian Medical Centre; Muhimbili University College of Health Sciences: Muhimbili University of Health and Allied Sciences; Muhimbili University College of Health Sciences: Muhimbili University of Health and Allied Sciences; Tanga Regional Hospital; Muhimbili University College of Health Sciences: Muhimbili University of Health and Allied Sciences; Mnazi Mmoja Referral Hospital; Duke Global Health Institute; Duke University School of Medicine; Duke Global Health Institute 0000-0002-7396-4789

**Keywords:** Implementation Science, Sustainability, Normalization Process Theory, emergency department, LMICs, Tanzania, sub-Saharan Africa, myocardial infarction

## Abstract

**Background:**

The Multicomponent Intervention to Improve Acute Myocardial Infarction Care (MIMIC) was developed to address gaps in AMI diagnosis and treatment in northern Tanzania. Although initial implementation was promising, many quality improvement interventions are not sustained after research support ends, especially in resource-limited settings. Evaluating sustainability and normalization is essential for understanding the long-term impact of implementation research. We evaluated these outcomes for the MIMIC intervention in a Tanzanian emergency department following a pilot implementation trial.

**Methods:**

We conducted a cross-sectional survey of all full-time emergency department clinicians (n = 35) at Kilimanjaro Christian Medical Centre (KCMC) using two validated implementation science tools: the Clinical Sustainability Assessment Tool (CSAT) and the Normalization MeAsure Development (NoMAD) questionnaire. The CSAT assesses seven domains, with higher scores reflecting greater perceived sustainability. The NoMAD measures four constructs, with higher scores indicating stronger normalization. For each domain, scores were summarized descriptively (means, standard deviations) and compared by provider type (doctors vs. registered nurses) using independent t-tests.

**Results:**

All 35 eligible clinicians (100%) completed the survey. Mean CSAT domain scores ranged from 5.81 (SD 1.04) for *Organizational Context and Capacity* to 6.73 (SD 0.47) for *Outcomes and Effectiveness* (scale 1–7). Mean NoMAD scores were uniformly high and clustered within a narrow range from 4.26 (SD 0.51) for *Collective Action* to 4.69 (SD 0.42) for *Cognitive Participation* (scale 1–5). Nurses reported significantly greater *Workflow Integration* than doctors (mean 6.76 vs. 6.20, p = 0.034); no other domains differed significantly by provider type. Domains related to perceived clinical benefit, individual engagement, and feedback scored highest, whereas organizational context and financial support scored comparatively lower.

**Conclusions:**

This study is among the first to apply the CSAT and NoMAD tools to evaluate a quality improvement intervention in sub-Saharan Africa. Findings indicate that MIMIC is both highly sustainable and normalized in routine care at KCMC, as reflected by consistently high mean domain scores across both instruments, although formal thresholds for these measures have not yet been established. Strengthening organizational capacity and long-term support, particularly financing and team coordination, may further enhance sustained implementation.

## Background

Acute myocardial infarction (AMI) is a leading cause of death globally, contributing to an estimated 3 million deaths annually.(1) While high-income countries have achieved significant reductions in AMI mortality through early diagnosis and use of evidence-based treatments,(2,3) low- and middle-income countries—which now account for over 80% of global cardiovascular disease deaths—continue to face major challenges in AMI recognition and care.(4)

In Tanzania, AMI is frequently under-diagnosed and under-treated. In a northern Tanzanian emergency department (ED), we found that nearly 90% of AMI cases are missed during routine care, and fewer than 25% of patients with AMI receive recommended treatments such as aspirin.(5–7) These gaps in care likely contributed to a 30-day mortality rate of 43% among AMI patients—one of the highest AMI mortality rates ever reported worldwide.(6)

To address these challenges, we developed the Multicomponent Intervention to Improve Acute Myocardial Infarction Care (MIMIC). Adapted from Brazil’s ACS-BRIDGE program and contextualized for the northern Tanzanian setting using the ADAPT-ITT framework,(7,8) MIMIC was evaluated in a one-year, single-arm pilot trial at Kilimanjaro Christian Medical Centre (KCMC) in northern Tanzania. The intervention led to substantial improvements in key care metrics, including rates of ECG and troponin testing, AMI identification, and evidence-based treatment with aspirin, clopidogrel, and heparin.(9–11)

While findings were encouraging, many research-driven quality improvement interventions are not sustained after the study period ends due to challenges like limited institutional support, staff turnover, and poor integration into daily workflows.(12) Sustaining interventions is difficult even in high-resource settings; for example, one review found that a third of quality improvement projects in the UK National Health Service were not maintained in real-world clinical settings after one year.(13) Poor sustainability of quality improvement interventions can lead to diminished care quality, worse patient outcomes, and inefficient use of both financial and non-financial resources.(14,15) These concerns underscore the need to evaluate not only short-term outcomes of interventions but also whether interventions demonstrate long-term sustainability and become embedded in routine clinical practice. Indeed, a growing number of implementation scientists have emphasized the importance of assessing sustainability and normalization of interventions in real-world clinical settings, beyond short-term implementation-effectiveness trials.(16)

We aimed to evaluate the longer-term sustainability and normalization of MIMIC following the conclusion of the one-year pilot trial. To do so, we conducted a follow-up survey among providers at KCMC using two validated implementation science tools: the Clinical Sustainability Assessment Tool (CSAT) and the Normalization MeAsure Development (NoMAD) questionnaire. CSAT measures an organization’s capacity to sustain interventions across seven domains, including leadership support, workflow fit, and performance monitoring.(17) NoMAD, based on Normalization Process Theory, assesses the extent to which an intervention becomes embedded in practice through constructs such as coherence, cognitive participation, collective action, and reflexive monitoring.(18) By administering CSAT and NoMAD, we sought to determine whether the intervention remained in use and to identify factors that supported or limited its continued implementation.

## Methods

### Setting

This study was conducted at KCMC, a 630-bed tertiary referral hospital located in Moshi, northern Tanzania. KCMC serves a catchment area of over 15 million people and includes an ED that provides 24-hour acute care. The ED is staffed by a team of nurses and doctors in a high-volume, resource-limited environment. Local challenges such as staffing variability, high clinical workload, and infrastructural constraints may affect the long-term sustainability of quality improvement efforts.

### The MIMIC Intervention

The MIMIC intervention was a multicomponent strategy designed to improve diagnosis and treatment of AMI in a low-resource emergency care setting. It included five core components: (1) a triage card placed on the stretchers of patients with potential AMI symptoms to prompt doctor consideration of the diagnosis; (2) a pocket reference card outlining evidence-based AMI care steps; (3) a web-based refresher training module on AMI diagnosis and treatment, required for all ED clinicians; (4) educational materials for patients, including printed pamphlets and visual messaging displayed in the ED waiting room; and (5) the appointment of doctor and nurse “champions” responsible for encouraging intervention uptake and coordinating implementation. All components were developed and refined using stakeholder input and context-specific adaptation and were delivered by KCMC ED staff during routine clinical care.(19)

The MIMIC pilot trial was conducted at KCMC between September 1st, 2023, and August 31st, 2024. During the pilot trial, MIMIC was implemented by the KCMC ED staff; given the positive results of the pilot trial,(9–11) the ED staff decided to continue implementing MIMIC as part of routine ED care.

### Participant Selection

All full-time doctors and registered nurses employed in the KCMC ED between November 2024 and May 2025 were eligible to participate. Clinicians were included regardless of prior involvement in the MIMIC pilot trial, provided they were employed full-time in the ED at the time of survey distribution. At the time of the survey, the KCMC ED employed 18 full-time nurses and 17 full-time doctors.

### Study Procedures

Participants were approached in person at work by a member of the research team during break periods. A brief explanation of the study’s purpose and procedures was provided. Participation was voluntary, and written informed consent was obtained prior to survey administration. The survey was anonymous and self-administered on a tablet to minimize social desirability bias. All survey questions were provided in both English and Swahili. Participants received 5,000 Tanzanian shillings (approximately 2 USD) as compensation for their time. Completed surveys were stored in a secure, password-protected database accessible only to the research team.

### Survey

The survey combined CSAT and NoMAD, two widely used implementation science tools with strong reliability and construct validity.(17,20,21) CSAT has been applied in resource-limited hospital settings, (21) while NoMAD has been used across diverse healthcare contexts to assess normalization.(20)

We administered the validated 21-item short version of the CSAT to minimize respondent burden while maintaining comprehensive assessment.(22) The tool includes 21 items across seven domains: engaged staff and leadership, organizational readiness, workflow integration, implementation and training, monitoring and evaluation, outcomes and effectiveness, and infrastructure.(17,22) Items were rated on a 7-point Likert scale ranging from “not at all” (score of 1) to “to a great extent” (score of 7), with an optional “don’t know” response. These domains were used to assess the ED’s capacity to sustain MIMIC over time.

NoMAD includes 20 items aligned with four constructs from Normalization Process Theory: coherence, cognitive participation, collective action, and reflexive monitoring.(18) Items were rated on a 5-point Likert scale from “strongly disagree” (score of 1) to “strongly agree” (score of 5) with optional “not relevant” and “don’t know” responses. These items were used to evaluate the extent to which MIMIC had become embedded in routine clinical practice.

Six supplementary questions addressed participants’ roles, clinical experience, prior involvement in MIMIC, and perceived ability to influence ED workflows. The survey took approximately 15 minutes to complete. The full instrument is included as Additional file 1.

### Statistical Methods

Survey responses were summarized using descriptive statistics. Total and domain-level scores for the CSAT and NoMAD were reported as means and standard deviations. Although Likert-scale data are ordinal, responses were treated as continuous to facilitate comparison across domains and constructs, consistent with prior studies using these instruments. (20,23–25) Accordingly, CSAT scores were averaged within each of the seven domains to assess organizational capacity for sustainability.(17)

NoMAD responses were similarly averaged within four constructs based on Normalization Process Theory.(20) Responses marked as “unable to answer” or “not relevant” were excluded from analysis. A total of 9 CSAT responses (1.2%) and 1 NoMAD response (0.1%) were excluded for this reason.

To ensure scoring consistency in the NoMAD tool, the item *“The MIMIC intervention disrupts working relationships”* (commonly listed as Item 10) was reverse coded so that higher scores indicate greater normalization, consistent with the directionality of other items.

To evaluate differences by provider type (doctor vs. nurse), independent-samples t-tests were conducted for overall CSAT and NoMAD scores, as well as for each individual domain score within both tools. For the purposes of analysis, providers were categorized into two groups: “doctor” (including both general and emergency specialist doctors) and “nurse” (including all registered nurses. A p-value of less than 0.05 was considered significant statistically. Given the use of CSAT and NoMAD in a novel population from a low-resource emergency setting, we assessed the internal consistency of each instrument and its individual domains in our setting using Cronbach’s alpha.

Statistical analyses were performed using R Statistical Software (version 4.5.1; R Core Team 2024).

### Ethics

Ethical approval for this follow-up study was obtained from the Tanzania National Institute for Medical Research (NIMR/HQ/R.8a/Vol. IX/2436), Kilimanjaro Christian Medical Centre (Proposal 893), and the Duke Health Institutional Review Board (Pro00090902). All procedures adhered to the ethical principles outlined in the Declaration of Helsinki (2000 revision). Written informed consent was obtained from all participants prior to survey administration. Materials were available in both English and Swahili to ensure participant understanding, and participation was voluntary. Respondents could decline or withdraw at any time without penalty.

### Reporting guidelines

This manuscript was prepared in accordance with the StaRI checklist for reporting implementation studies and the STROBE checklist for observational studies. The completed checklists are provided in the Supplementary Materials (Additional file 2 and Additional file 3).

## Results

### Participant Characteristics

All 35 emergency department clinicians completed the survey, including 18 nurses (51%), 15 general doctors (43%), and 2 emergency specialist doctors (6%). The mean age was 32.7 years (SD 6.9), and participants reported an average of 3.4 years (SD 2.7) of clinical experience. Most participants (n = 29, 83%) reported delivering the MIMIC intervention as part of their routine clinical duties, while the remaining six (17%) served as champions or supervisors (Table 1).

### CSAT Scores

**Figure 1:**
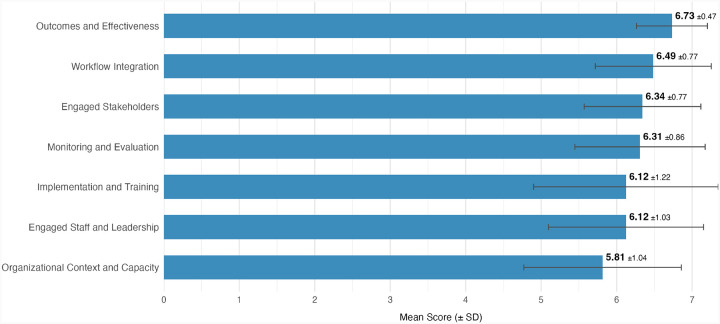
CSAT Domain Scores All items are scored 1 to 7; higher values represent stronger sustainability capacity in that domain.

CSAT domain scores, rated on a 7-point Likert scale, indicated high perceived capacity to sustain the intervention. Scores ranged from 5.81 (SD 1.04) for *Organizational Context and Capacity* to 6.73 (SD 0.47) for *Outcomes and Effectiveness* (Table 2; Fig. 1). Item-level response distributions are shown in Additional file 4.

#### NoMAD Scores

NoMAD domain scores, rated on a 5-point Likert scale, reflected strong normalization of the MIMIC intervention into routine clinical practice. Scores were highest for Cognitive Participation (mean 4.69, SD 0.42) and Reflexive Monitoring (mean 4.50, SD 0.43), followed by Coherence (mean 4.46, SD 0.55) and Collective Action (mean 4.26, SD 0.51). Item-level response distributions are shown in Additional file 5.

**Figure 2:**
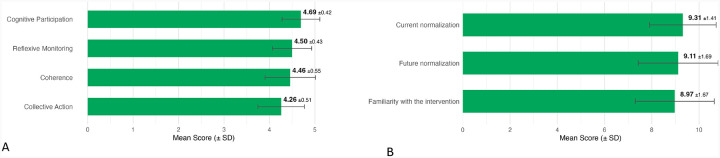
Summary of NoMAD Scores. Panel A. NoMAD Domain Scores All items are scored from 1 to 5; higher values represent greater normalization for that subscale. The item “disrupts working relationships” (Collective Action domain) was reverse coded so that higher scores reflect greater normalization. Panel B. General Normalization Item Scores All items are scored from 1 to 10. For “familiarity,” higher scores indicate the intervention feels more familiar. For “current” and “future” normalization, higher scores indicate greater perceived normalization.

Three general normalization items, rated on a 10-point Likert scale, were analyzed separately in accordance with prior literature.(20,23) Participants reported high familiarity with the MIMIC intervention (mean 8.97, SD 1.67), perceived it to be well normalized in current practice (mean 9.31, SD 1.41), and anticipated continued normalization in the future (mean 9.11, SD 1.69) (Fig. 2, Panel B). Full distributions are presented in Additional file 6.

### Internal Consistency

Internal consistency of domain scores was assessed using Cronbach’s. Among CSAT domains, alphas ranged from 0.54 to 0.83 (overall = 0.91). Among NoMAD domains, alphas ranged from 0.61 to 0.84 (overall = 0.89). Cronbach’s alpha values for the full instrument and each domain are presented in Table 2.

### Provider Comparison

Nurses rated Workflow Integration significantly higher than doctors (mean 6.76 vs. 6.20, *p* = 0.034). No other CSAT domains differed significantly by provider type. NoMAD domain scores and general normalization items (familiarity, current and future normalization) also showed minimal variation between doctors and nurses (Table 3).

## Discussion

Our findings suggest that MIMIC is both sustainable and normalized in routine emergency care at KCMC. High scores across CSAT and NoMAD domains reflect strong perceived organizational capacity and widespread normalization among providers. Domains related to perceived clinical benefit, compatibility with existing workflows, and individual engagement scored especially well, underscoring that MIMIC continues to be seen as valuable, aligned with clinical priorities, and well-integrated into clinical routines.

Several features of the MIMIC intervention likely contributed to these high scores. Its iterative, participatory design involving frontline KCMC providers ensured alignment with local workflows and responsiveness to site-specific barriers to AMI care.(19) Low-cost, intuitive tools—such as color-coded triage cards, pocket reference guides, and discharge checklists—were supported by visible clinical reminders and weekly case-based audits, enhancing provider engagement and reinforcing practice change. The intervention also emphasized shared responsibility between nurses and doctors, with designated champions from both cadres auditing AMI care and ensuring implementation of all MIMIC components.(19) Collectively, these characteristics—workflow fit, perceived clinical value, collective ownership, and continuous feedback—align closely with CSAT and NoMAD constructs and likely underlie the strong perceptions of MIMIC as both sustainable and normalized in routine care.

While most domains showed minimal variation by provider type, nurses rated workflow integration higher than doctors. This may reflect their central role in delivering key components of the intervention—triaging patients for AMI symptoms, distributing educational materials, and reinforcing clinical reminders at the bedside—as well as greater day-to-day exposure to MIMIC-related activities. Differences in responsibilities and proximity to specific implementation tasks may shape how integrated the program feels to different provider groups.

Despite overall strong results, lower scores in domains tied to organizational context and team-level coordination highlight areas for improvement. Item-level responses in these domains showed greater variability and more neutral ratings, particularly regarding perceived availability of financial resources, adequacy of training, and alignment of task assignments with staff skills. Notably, the items with the fewest respondents strongly agreeing on both the CSAT and NoMAD pertained to financial resources. The relevant CSAT item encompassed time, space, and funding needed to achieve intervention goals; responses to the NoMAD item regarding the availability of “sufficient resources” were similarly mixed. These findings echo challenges observed during the pilot trial—staffing variability, resource constraints, and gaps in coordination across roles—and point to modifiable barriers.(9–11) Targeted strategies such as strengthened leadership engagement, clearer delineation of team-based roles, and enhanced interprofessional training may help bolster institutional support and promote long-term sustainability. Given that the full cost of the MIMIC intervention reported in the initial MIMIC pilot trial was 1324 USD annually, most of which was attributed to champion stipends,(26) securing additional funding or exploring non-monetary means to support the champions may further bolster long-term sustainability and normalization of the intervention.

The overall domain-level patterns observed in our study are consistent with prior studies using CSAT and NoMAD.(17,21,27–29) Across contexts, CSAT domains assessing leadership support, perceived benefit, and workflow integration often score highest, while organizational infrastructure and team coordination show greater variability, particularly in resource-limited environments..(17,21,29) Similarly, NoMAD evaluations in low-resource settings mirror our findings: the PACE program in Tanzania reported strong provider engagement and feedback mechanisms but lower scores in team coordination due to staffing and supply constraints.(28) A dementia care study likewise found high provider buy-in but emphasized infrastructure and interprofessional coordination as critical to sustainability.(27) These parallels reinforce that sustained normalization depends on both integration into clinical routines and broader organizational support.

We found high Cronbach’s alpha values for both CSAT and NoMAD, indicating strong reliability and internal consistency. These results closely mirror the original validation findings reported by Malone et al. (2021) and Finch et al. (2020),(17,20) which were conducted in the United States and the United Kingdom, respectively. As is typical for multidimensional implementation measures, domains with fewer or conceptually diverse items (e.g., Organizational Context and Capacity; Reflexive Monitoring) yielded lower alpha values, while the full scales demonstrated robust internal consistency.(22,23) The slightly lower CSAT alphas likely reflect the combination of short subscales with few items and the modest sample size (n = 35), both of which are known to attenuate reliability estimates.(30,31) Overall, these results support the reliability of both instruments for assessing sustainability and normalization in the Tanzanian healthcare context.

To our knowledge, this study represents one of the earliest applications of the CSAT and NoMAD instruments in emergency medicine and among the first efforts to apply them in implementation research in sub-Saharan Africa. Use of these instruments proved feasible, relevant, and informative in our setting, as demonstrated by complete participation from eligible clinicians and consistent, interpretable responses across both tools. Researchers conducting implementation work elsewhere in the region should consider these tools to assess long-term sustainability and normalization—two often-overlooked outcomes, particularly in resource-limited acute care settings where such data remain scarce.

This study had several strengths. First, it assessed sustainability after active implementation support ended, offering rare insight into post-trial intervention persistence. Second, the combined use of CSAT and NoMAD provided a complementary evaluation of current integration and future sustainability capacity. Third, inclusion of both nurses and doctors allows for comparison across provider groups, and the 100% response rate (35 of 35) enhances internal validity and minimizes response bias.

Several limitations should also be noted. First, while CSAT and NoMAD capture key dimensions of implementation processes, they are best interpreted alongside complementary data sources. Prior work highlights the value of mixed-methods approaches—such as qualitative interviews or direct observation—to capture contextual nuances.(23,32) A qualitative study exploring provider perspectives on long-term MIMIC sustainability is ongoing and will be published separately. Second, the cross-sectional design limits our ability to assess temporal trends or infer causality; longitudinal data may better characterize how normalization evolves in response to staffing changes or workflow adaptations. Third, the study was conducted at a single emergency department with a modest sample size, limiting generalizability.

Fourth, social desirability bias may have skewed responses toward favorable answers. However, independent, tablet-based survey administration likely reduced this bias and facilitated candid responses. Finally, neither the CSAT nor NoMAD has been evaluated to determine whether higher scores predict future sustainability or normalization;(16,24) this lack of established predictive validity is a limitation of both the instruments and our study. Despite this, the high response rate and consistent domain-level patterns in our study suggest that these instruments captured meaningful provider perceptions, which are important precursors to sustained adoption.(29) Ongoing analyses of MIMIC’s long-term impact on AMI care delivery will help confirm their predictive validity, addressing a key evidence gap in implementation science.(16,24)

Future research should evaluate the long-term clinical impact of MIMIC, its potential for national scale-up, and system-level strategies to support long-term adoption. Repeat assessments of sustainability and normalization—ideally at multiple time points—may help identify key inflection points and guide adaptive implementation support. Incorporating sustainability planning into routine operational processes may further support long-term integration.

## Conclusion

The MIMIC intervention remained in active use and was perceived by clinicians as both sustainable and normalized within emergency care workflows at KCMC following the conclusion of the pilot trial. These findings demonstrated the feasibility of sustaining a multicomponent intervention in a resource-limited setting when it aligned with clinical priorities and was reinforced by ongoing engagement. This study highlighted the utility of structured tools like CSAT and NoMAD for assessing early sustainment and normalization, particularly in low-resource acute care environments. Insights from this evaluation may inform efforts to strengthen the durability of similar interventions across LMIC health systems.

## Supplementary Material

This is a list of supplementary files associated with this preprint. Click to download.

- Additionalfiles.docx
- Additionalfile1.xlsx
- Additionalfile2.docx
- Additionalfile3.docx
- Additionalfile4.png
- Additionalfile5.png
- Additionalfile6.png

## Figures and Tables

**Table 1:** Participant Characteristics

Measure	*N (%) or Mean (SD)* ^1^
Gender	Female: 14 (40%)
	Male: 21 (60%)
Age (years)	32.7 (6.9)
Years of Clinical Experience	3.4 (2.7)
Role in MIMIC	Delivers MIMIC during routine ED work: 29 (83%)
	Supervises MIMIC (Champion/Supervisor): 6 (17%)
Provider Type	Emergency specialist physician: 2 (6%)
	General physician: 15 (43%)
	Nurse: 18 (51%)

1 Values are expressed as means (SD) for continuous variables or N (%) for categorical variables.

**Table 2:** CSAT and NoMAD Cronbach’s alpha by domain

Domain	Cronbach’s alpha
**CSAT**	
Engaged Staff and Leadership	0.67
Engaged Stakeholders	0.61
Organizational Context and Capacity	0.54
Workflow Integration	0.83
Implementation and Training	0.75
Monitoring and Evaluation	0.68
Outcomes and Effectiveness	0.70
Overall CSAT	0.91
**NoMAD**	
Coherence	0.84
Cognitive Participation	0.83
Collective Action	0.72
Reflexive Monitoring	0.61
Overall NoMAD	0.89

**Table 3:** CSAT and NoMAD Scores: Comparison of Nurse vs. Doctor Responses

Domain^1^	Doctor^2^ Mean (SD)	Nurse Mean (SD)	p-value
CSAT			
Engaged Staff and Leadership	6.12 (0.99)	6.13 (1.09)	0.973
	6.24 (0.92)	6.44 (0.62)	0.438
Organizational Context and Capacity	5.73 (1.04)	5.9 (1.07)	0.632
Workflow Integration	6.2 (0.93)	6.76 (0.45)	0.034*
Implementation and Training	5.9 (1.37)	6.33 (1.06)	0.308
Monitoring and Evaluation	6.06 (1.09)	6.55 (0.5)	0.106
Outcomes and Effectiveness	6.65 (0.61)	6.81 (0.29)	0.311
Overall CSAT Score	6.12 (0.87)	6.42 (0.47)	0.231
NoMAD			
Coherence	4.4 (0.56)	4.51 (0.56)	0.541
Cognitive Participation	4.59 (0.48)	4.79 (0.32)	0.157
Collective Action	4.16 (0.6)	4.35 (0.41)	0.286
Reflexive Monitoring	4.51 (0.46)	4.49 (0.41)	0.910
Overall NoMAD Score	4.38 (0.46)	4.51 (0.36)	0.369
General Normalization			
Familiarity with MIMIC	9.18 (1.47)	8.78 (1.86)	0.486
Current Normalization	9.24 (1.48)	9.39 (1.38)	0.753
Future Normalization	9.18 (1.51)	9.06 (1.89)	0.835

1 Values are expressed as means (SD) for continuous variables or N (%) for categorical variables.

2 Doctor group includes both general and emergency specialist physicians.

* *p* < 0.05

## Data Availability

The datasets used and/or analyzed during the current study are available from the corresponding author on reasonable request.

## Abbreviations

AMI: Acute Myocardial Infarction
CSAT: Clinical Sustainability Assessment Tool
ED: Emergency Department
KCMC: Kilimanjaro Christian Medical Centre
LMICs: Low–and Middle–Income Countries
MIMIC: Multicomponent Intervention to Improve Acute Myocardial Infarction Care
NoMAD: Normalization MeAsure Development questionnaire
NPT: Normalization Process Theory
USD: United States Dollar
